# Supplementary figures and images for: Embedding muscle fibers in hydrogel improves viability and preserves contractile function during prolonged ex vivo culture
Source: J Gen Physiol. 2025 Nov 3;158(1):e202513761. doi: 10.1085/jgp.202513761 (PMC12581944; doi:10.1085/jgp.202513761)

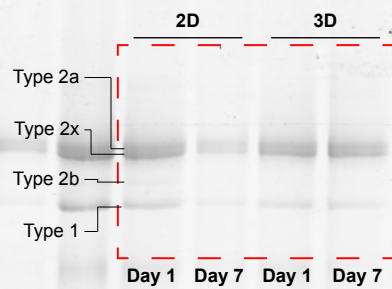

Supplement: SourceData F3 — is the source file for Fig. 3. [file jgp_202513761_sourcedataf3.pdf]

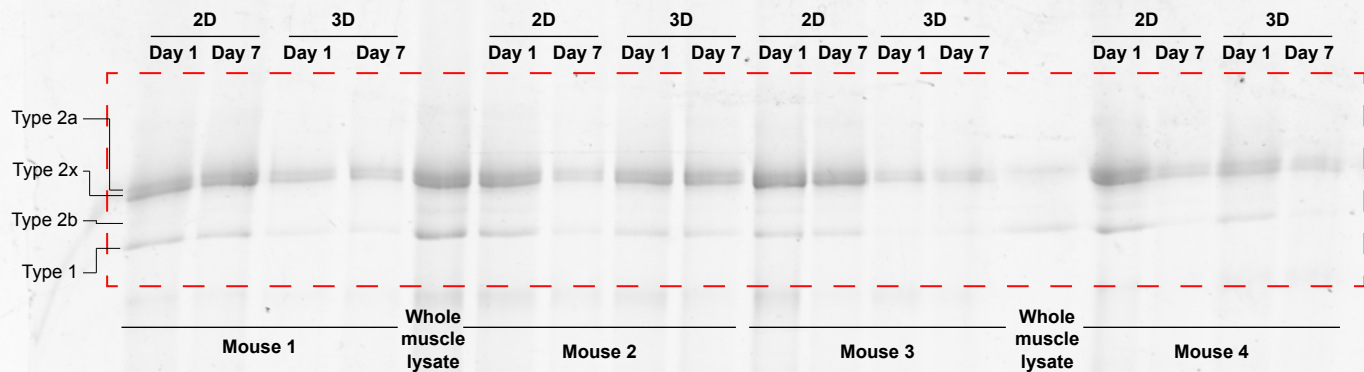

Supplement: SourceData FS6 — is the source file for Fig. S6. [file jgp_202513761_sourcedatafs6.pdf]
